# Human astrovirus draft genomes from under-five children presenting with gastroenteritis in Malawi from 2012 to 2024

**DOI:** 10.1128/mra.01289-25

**Published:** 2026-04-27

**Authors:** Ernest Matambo, Flywell Kawonga, Chimwemwe Mhango, End Chinyama, Josephine Msowoya, Clara Majengo, Ayodeji E. Ogunbayo, Nkosazana Shange, Hlengiwe Sondlane, Milton T. Mogotsi, Francis E. Dennis, Prisca Benedicto Matambo, Arox W. Kamng'ona, Benjamin Kumwenda, Martin M. Nyaga, Celeste M. Donato, Chrispin Chaguza, Khuzwayo C. Jere

**Affiliations:** 1 Department of Pharmacy, School of Life Sciences and Allied Health Professions, Kamuzu University of Health Sciences 37610, Blantyre, Malawi; 2 Malawi-Liverpool-Wellcome Programme, Queen Elizabeth Central Hospital Campus 560808, Blantyre, Malawi; 3 Institute of Infection, Veterinary and Ecological Sciences, University of Liverpool 4591, Liverpool, United Kingdom; 4 Department of Medical Laboratory Sciences, School of Life Sciences and Allied Health Professions, Kamuzu University of Health Sciences 37610, Blantyre, Malawi; 5 Biomedical Sciences Department, School of Life Sciences and Allied Health Professions, Kamuzu University of Health Sciences 37610, Blantyre, Malawi; 6 Next Generation Sequencing Unit, School of Biomedical Sciences and Division of Virology, Faculty of Health Sciences, University of the Free State 37702, Bloemfontein, South Africa; 7 Department of Electron Microscopy and Histopathology, Noguchi Memorial Institute for Medical Research, University of Ghana, Legon 58835, Accra, Ghana; 8 Enteric Diseases Group, Murdoch Children's Research Institute, Parkville 34361, Melbourne, Australia; 9 Department of Paediatrics, The University of Melbourne 2281, Parkville, Victoria, Australia; 10 Department of Host-Microbe Interactions, St Jude Children's Research Hospital 5417, Memphis, USA; 11 NIHR Mucosal Pathogens Research Unit, Division of Infection and Immunity, University College London 4919, London, United Kingdom; 12 NIHR Health Protection Research Unit in Gastrointestinal Infections, University of Liverpool 4591, Liverpool, United Kingdom; Katholieke Universiteit Leuven, Leuven, Belgium

**Keywords:** astrovirus, gastroenteritis, Malawi, draft genomes

## Abstract

Human astroviruses (HAstVs) account for 2% to 9% of gastroenteritis cases in children and are occasionally associated with central nervous system complications. We report six draft HAstV genomes from Malawian children <5 years old with gastroenteritis, providing baseline information for the understanding of HAstV diversity and disease burden in sub-Saharan Africa.

## ANNOUNCEMENT

Human astroviruses (HAstV) gastroenteritis (GE) is often self-limiting and has a global prevalence of 2%–9% in all age groups (1–3). In rare cases, children and the elderly may develop severe gastrointestinal, respiratory tract, and central nervous system infections, which may be fatal (3–7). GE is primarily linked to the classical HAstV types 1–8, which belong to the *Mamastrovirus hominis* species (genus: *Mamastrovirus*) of the *Astroviridae* family. In contrast, severe disease and extra-gastrointestinal infections are associated with Melbourne (MLB 1–3) and Virginia (VA 1–4)/human-mink-ovine (HMO A-C) types (*Mamastrovirus melbournense* and *Mamastrovirus virginiaense* species, respectively) (5, 8–10). The ~6 to 8 kb positive-sense, single-stranded RNA genome has three reading frames: ORF1a, ORF1b, and ORF2, which encode a protease, an RNA-dependent RNA polymerase, and the capsid protein, respectively (4, 11). In Malawi, Southeast Africa, whole-genome sequencing of HAstV had not been performed previously. We randomly selected 10 stool samples monthly (*n* = 960) from children <5 years old with gastroenteritis between 2012 and 2024 at Queen Elizabeth Central Hospital and Bangwe Health Center for HAstV detection and sequencing. Here, we report 6 of the 23 HAstV-positive RNA extracts that were sequenced, assembled, and had complete ORFs.

Total RNA was extracted from the stool specimen using the QIAamp Fast DNA Stool Mini Kit (Qiagen, Hilden, Germany) following the manufacturer’s protocol. HAstV was detected using real-time PCR with custom-designed enteric TaqMan Array Cards following the protocol as previously described (12, 13). The QIAamp RNA Mini Kit (Qiagen) was used to re-extract the astrovirus-positive stool samples, and the resulting RNA was quantified using the Qubit RNA HS Assay Kit (Thermo Fisher Scientific, USA). The cDNA was generated through reverse transcription with random primers followed by amplification using the QIASeq FX SC RNA Library Preparation Kit (Qiagen). Genome libraries were prepared using the Illumina DNA Prep Kit (c, USA), and sequencing was performed on the Illumina NextSeq 2000 platform using the P1 flow cell and 300-cycle reagent kit to produce 2 × 150 bp paired-end reads.

**Fig 1:**
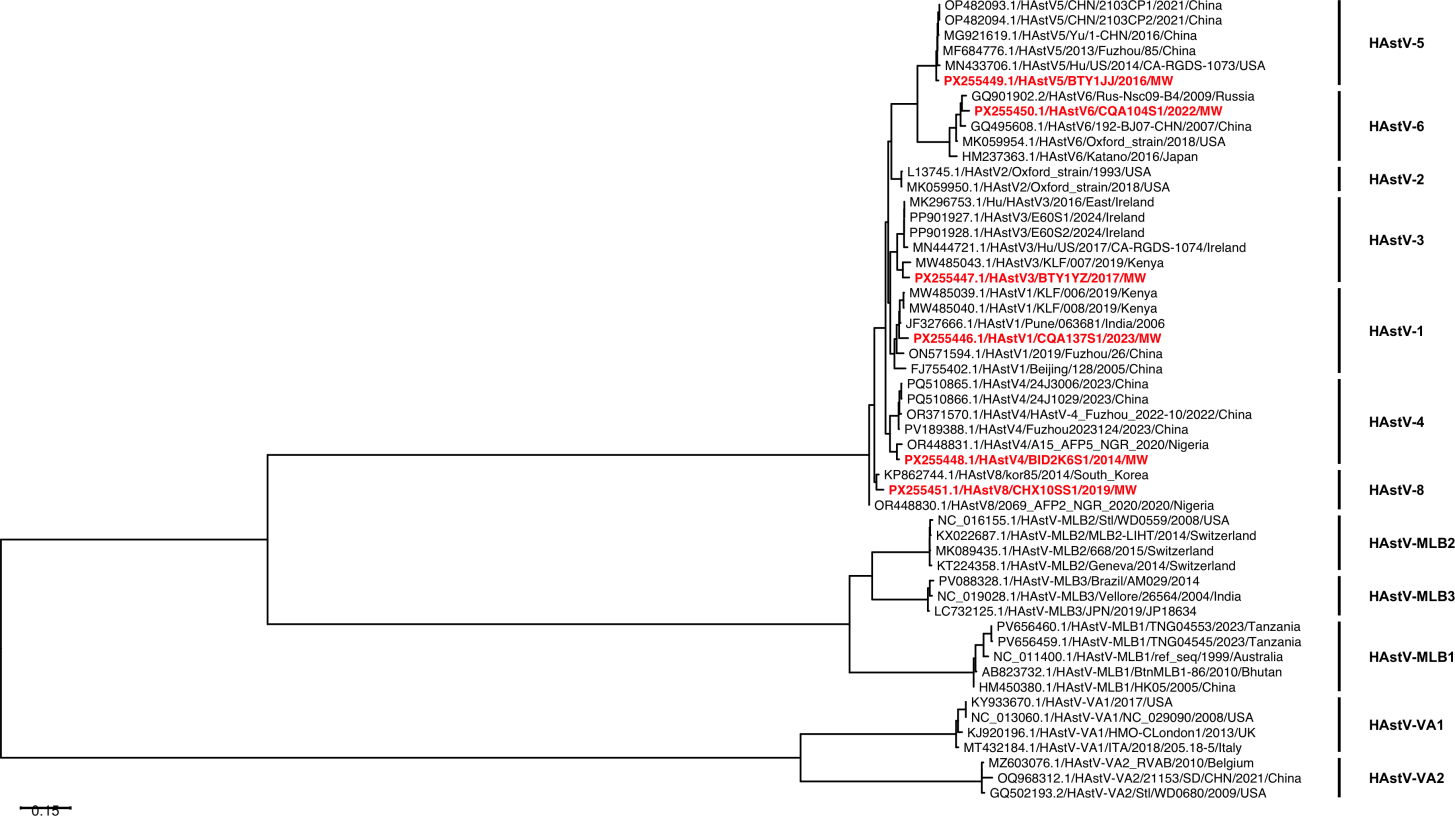
A maximum likelihood phylogeny showing the placement of the HAstV ORF1b sequences from Malawi (red taxon labels) in the context of the HAstV ORF1b sequences obtained from GenBank (accessed on 7 October 2025) as the ORF1b is a conserved region (24). The Malawian and NCBI HAstV reference genomes were aligned using MAFFT v7 (25). We reconstructed the phylogeny using IQ-TREE v2.3.0 with 1,000 bootstraps and rooted it at the midpoint of the branch separating the two most divergent taxa. We visualized it in R using the ggtree package v3.14.0 (https://www.r-project.org/) (26). Clustering of the Malawian sequences with published genomes confirmed their taxonomic and HAstV type assignments.

We filtered out reads based on quality and mapping to the human host genome using Trimmomatic v0.40, FastQC v0.12.1, and Bowtie 2 v2.5.4 (14–16). We then mapped the resulting non-human reads against reference HAstV genomes (Table 1) using BWA v0.7.18-r1243-dirty, and generated consensus sequences using iVar 1.4.4 (17, 18). We confirmed the viral taxonomy of the consensus genomes using Genome Detective Tool v2.94 (19). We then aligned the consensus sequences with other HAstV genomes in the NCBI database to identify the HAstV strain types based on the top five matching hits. We annotated the consensus sequences using NCBI ORF–FINDER, which revealed three ORFs (20, 21). We assessed the genome completeness and identity of the consensus sequences using CheckV v1.0.3 and Needle V in EMBOSS v6.6.0.0 (http://emboss.open-bio.org/), respectively (22). The draft genomes were analyzed for recombination using the Recombination Detection Program v5.64 (23). Default parameters were used for each software. Table 1 summarizes the generated consensus genomes, while Fig. 1 displays the ORF1b-gene-based phylogeny of the Malawian strains in the context of other HAstV genomes obtained from GenBank. The generated consensus genomes demonstrated successful sequencing and assembly of astrovirus sequences from Malawi, enabling downstream analyses.

**TABLE 1:** Summary characteristics of the generated HAstV sequencing reads and consensus genomes^*a*^

Sample name	CQA137S1	BTY1YZ	BID2K6S1	BTY1JJ	CQA104S1	CHX10SS1
Sample type	Stool	Stool	Stool	Stool	Stool	Stool
Sample collection date (year-mo-day)	2023-Jun-15	2017-Jul-04	2014-Feb-06	2016-Mar-10	2022-Nov-23	2019-May-23
Ct values	31.21	34.44	32.52	33.679	31.75	33.48
Sequencing platform	Illumina NextSeq 2000	Illumina NextSeq 2000	Illumina NextSeq 2000	Illumina NextSeq 2000	Illumina NextSeq 2000	Illumina NextSeq 2000
Read type	Paired	Paired	Paired	Paired	Paired	Paired
Number of reads	829,360	1,132,097	1,860,216	1,880,100	1,657,104	208,315
Average read length (trimmed)	108.72	121.75	121.1	117.22	113.77	124.23
Average sequencing depth	1,116.2×	222.3×	657.6×	763.3×	3,743.1×	823.5×
Average sequencing coverage (%)	100	99.8	99.8	100.0	99.7	99.9
Reference genome accession	MW485040.1	MW485043.1	OR448831.1	MF684776.1	MK059954.1	KP862744.1
Consensus genome identity (%)^*b*^	95.9	93.6	96.5	96.7	94.4	97.3
GC (%)	51	49	48	51	53	46
Consensus genome length (nt)	6,775	6,695	6,768	6,738	6,719	6,765
ORF1a length (nt)	2,802	2,685	2,802	2,802	2,763	2,781
ORF1b length (nt)	1,107	1,476	1,107	1,110	1,110	1,110
ORF2 length (nt)	2,364	1,962	2,490	2,535	2,340	2,352
Consensus genome completeness (%)	100	100	100	100	99.99	100
HAstV type	1	3	4	5	6	8
Consensus genome accession numbers	PX255446.1	PX255447.1	PX255448.1	PX255449.1	PX255450.1	PX255451.1
SRA read accession numbers	SRX30791383	SRX30791384	SRX30791385	SRX30791386	SRX30791387	SRX30791388

^*a*^ Due to the diverse nature of the HAstV types, different reference genomes were used. No recombination was detected in the consensus sequences.

^*b*^ Consensus genome identity was calculated relative to the reference strains shown in the table.

## Data Availability

All reported genomes and their corresponding reads have been deposited in GenBank and the Sequence Read Archive databases, respectively, as shown in Table 1. The study was approved by the Malawi National Health Science Research Committee (NHSRC #867).
